# Early immunotherapy in a patient with myelin oligodendrocyte glycoprotein antibody-associated disease with syphilis and acquired immunodeficiency syndrome: a case report

**DOI:** 10.3389/fimmu.2025.1591365

**Published:** 2025-07-22

**Authors:** Qiu-Xia Zhang, Lin-Jie Zhang, Bin Zhao

**Affiliations:** Department of Neurology, Tianjin Neurological Institute, Tianjin Medical University General Hospital, Tianjin, China

**Keywords:** inflammatory, central nervous system, syphilis, HIV, MOGAD

## Abstract

This is the case of a 41-year-old man who presented with blurred vision, unconsciousness, and fever with a positive Babinski sign on both sides. Blurred vision, unconsciousness, and fever are commonly seen in neurology and emergency settings, with a wide range of potential etiologies. Myelin oligodendrocyte glycoprotein antibody-associated disease (MOGAD) often occurs after infection, but cases of syphilis and human immunodeficiency virus as precursors are rarely reported. We report a case of MOGAD with syphilis and acquired immunodeficiency syndrome. Additionally, the study summarizes the disease’s clinical manifestations, therapy, prognosis, and outcome and highlights the importance of timely and accurate immune regulation therapy.

## 1 Introduction

Myelin oligodendrocyte glycoprotein antibody-associated disease (MOGAD) is a rare antibody-mediated inflammatory demyelinating disorder (1). Studies have shown that the prevalence is 0.51–3.42 per 100,000 individuals, with incidences varying from 0.112 to 0.48 per 100,000 person-years (2). An autoimmune response to an infection may be a triggering factor for MOGAD, with possible mechanisms including molecular mimicry, epitope diffusion, and the activation of B cells. MOG antibodies cross the blood–brain barrier to bind to autoantigen expressed on myelin, resulting in secondary demyelination (3). Here, we report a case of MOGAD with syphilis and acquired immunodeficiency syndrome (AIDS) that presented with blurred vision, unconsciousness, and fever. Intravenous immunoglobulin (IVIG) significantly improved the symptoms of the patient. In clinical practice, various acute treatments such as high-dose intravenous methylprednisolone (IVMP), plasmapheresis, and IVIG are used for MOGAD. IVMP and plasmapheresis may negatively impact patients with immunodeficiency or active infection, such as those with syphilis or AIDS, whereas IVIG may offer both immunomodulatory and anti-infective benefits.

## 2 Case report

A 41-year-old man with a 3-year history of syphilis and AIDS presented to the hospital with sudden-onset blurred vision in both eyes lasting for 1 hour. The patient had no dysarthria, dysphagia, or cognitive changes. No abnormalities were noted in limb movements or sensations. Four hours after symptom onset, the patient experienced unconsciousness accompanied by fever. Initial neurological examination revealed blindness in his left eye and vision loss in the right eye, with the ability to count fingers only at a distance of 2 ft. The pupils were equal in size, measuring 5 mm bilaterally, with relative afferent pupillary defects on both sides. Other neurological examinations did not reveal any significant abnormalities. Over the next 4 hours, his temperature was 39.1°C, and he gradually developed a state of somnolence, with a positive Babinski sign on both sides. Other neurological findings were inconclusive.

Basic tests related to infection, immunity, inflammation, and cerebrovascular disease were individually performed. Workup revealed elevated C-reactive protein (35.32 mg/L), rheumatoid factor (20.70 IU/mL), anti-streptolysin O (207.00 IU/mL), antinuclear antibody (1:80), Ro52 antibody (positivity), human immunodeficiency virus (HIV) antibody (positivity), syphilis with serum toluidine red unheated serum test (TRUST) (1:2), and anti-*Treponema pallidum*-specific antibodies (positivity, 245.50 S/CO). Tests, including complete blood count, biochemical testing, and hepatitis antibodies, were all negative. The peripheral blood CD4+ T cells were 862 cells/μL, accounting for 38%. The lumbar puncture pressure was 224 mmH_2_O, and the cerebrospinal fluid (CSF) was colorless and transparent, with elevated white blood cells (30 × 10^6^/L) and protein (0.94 g/L). The glucose and chloride levels in the CSF were normal, and cultures and cryptococcal antigen tests were negative. TRUST in the CSF was negative, whereas anti-*T. pallidum*-specific antibodies were positive (47.51 S/CO). The MOG antibody (cell-based assay) in the serum and CSF was positive, with ratios of 1:320 and 1:10, respectively. Aquaporin-4 (AQP4) antibody, glial fibrillary acidic protein (GFAP) antibody, and oligoclonal bands in both the serum and CSF were all negative.

**Figure 1:**
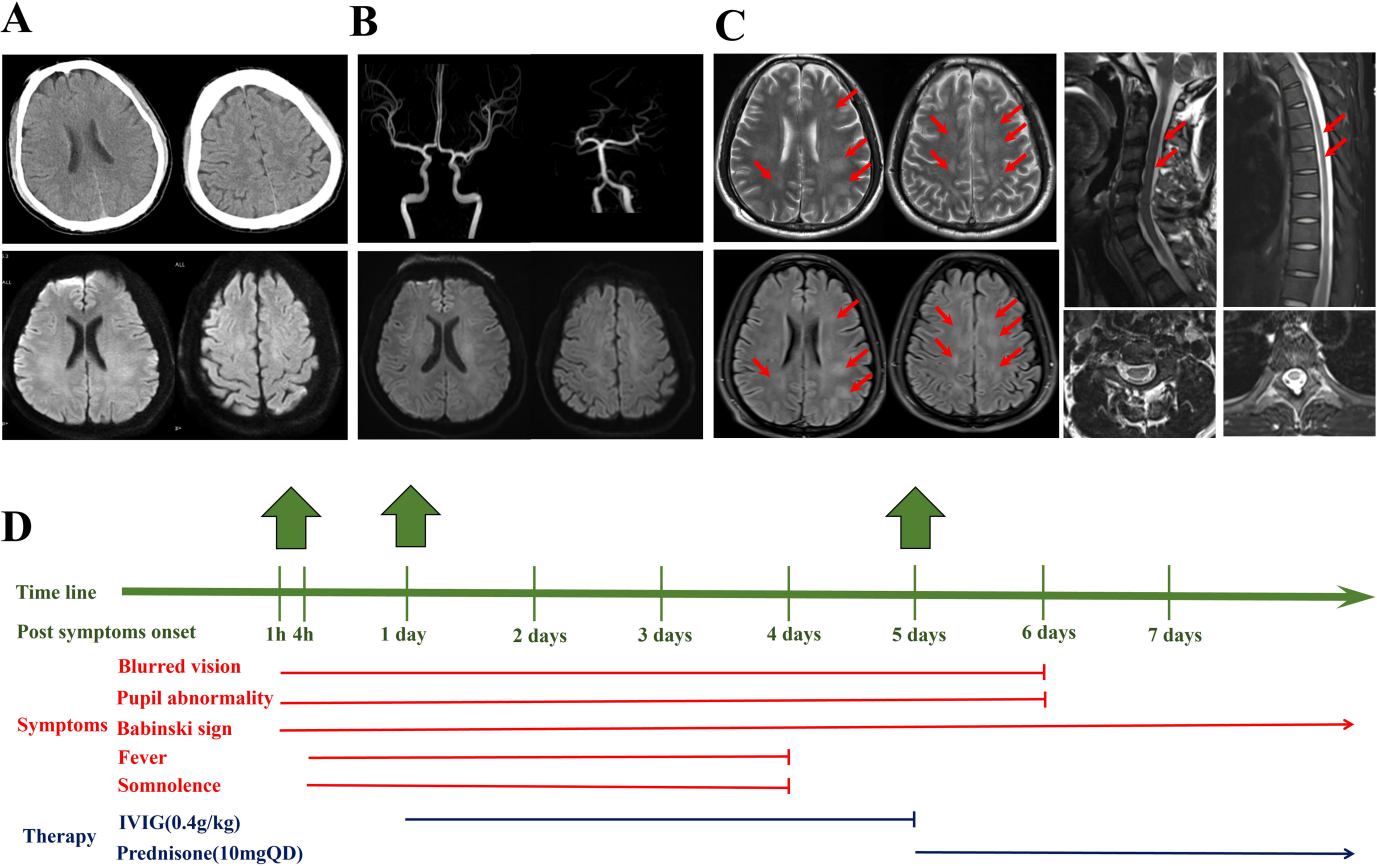
**(A)** Four hours after the onset of symptoms, the computed tomography and magnetic resonance imaging (MRI) of the brain did not reveal obvious bleeding, infarction, etc. **(B)** One day after onset, the magnetic resonance angiography of the brain was normal. **(C)** Five days after symptom onset, the brain MRI demonstrated multiple abnormal signals on the T2 and T2 FLAIR sequences, mainly affecting the white matter as indicated by the arrow. Abnormal signals can be observed in C2–C4 and T5–T7 in the spinal cord. **(D)** The timeline, clinical symptoms, and therapy of the patient.

The patient presented with acute blurred vision for 1 hour, and computed tomography (CT) and magnetic resonance imaging (MRI) of the brain demonstrated negative results for acute intracranial abnormalities. Brain magnetic resonance angiography (MRA) was normal. Five days after onset, brain, cervical, and thoracic spinal cord MRIs were performed, and the results revealed multiple abnormal white matter signals in the brain, as well as abnormal signals in the cervical and thoracic spinal cords (**Figure 1**).

One day after onset, intravenous immunoglobulins (0.4 g/kg × 5 days) were administered. The patient was discharged with oral prednisone (10 mg QD). The patient was followed up for 6 months after discharge, and prednisone was gradually reduced to 7.5 mg QD. The patient had no obvious neurological positive signs, except for a positive Babinski sign on both sides. Unfortunately, the patient refused MRI follow-up. During the follow-up, we found that the MOG antibody (cell-based assay) in the serum remained positive, with a ratio of 1:320.

## 3 Discussion

According to the MOGAD diagnostic criteria proposed by the International MOGAD Panel in 2023, rapid worsening of clinical deficits from onset to nadir within minutes to hours is recognized as a red flag of MOGAD. MOGAD with rapid progress and the combination of syphilis and AIDS are very rare. We carefully evaluated the case and concluded the diagnosis of MOGAD. First, the patient had optic neuritis (suspicious), myelitis, and acute disseminated encephalomyelitis (ADEM) as the core clinical demyelinating event. Second, in the acute phase, the MOG antibody was positive in the serum and CSF; in the remission phase, the MOG antibody in the serum remained positive (1:320). Third, the brain MRI demonstrated multiple abnormal signals on the T2 and T2 fluid attenuated inversion recovery (FLAIR) sequences, mainly affecting the white matter. We observed abnormal signals in C2–C4 and T5–T7 in the spinal cord. Fourth, we excluded diagnoses including neuromyelitis optica spectrum disorder (NMOSD), multiple sclerosis (MS), GFAP astrocytopathy, ADEM, central nervous system damage caused by neurosyphilis, and AIDS (**Table 1**). Unfortunately, the patient could not cooperate during the orbital MRI examination in both the acute and remission phases. Although the diagnosis of optic neuritis in the patient was questionable, other clinical manifestations, laboratory tests, and imaging supported the diagnosis of MOGAD.

**Table 1:** The differential diagnosis and key diagnostic features.

Classification	MOGAD	NMOSD	MS	ADEM	Neurosyphilis	AIDS
Age of onset, years	Children > adults	20–40 years	20–30 years	Children > adults	40–50 years	15–24 years; >60 years
Clinical manifestations						
Fever	Possible	Possible	Possible	Possible	Possible	Possible
Blurred vision	Possible	Possible	Possible	Possible	Possible	Possible
Unconsciousness	Possible	Possible	Possible	Possible	Possible	Possible
Laboratory test						
Elevated proteins in CSF	Possible	Possible	Possible	Possible	Possible	Possible
WBC pleocytosis in CSF	Possible	Possible	Possible	Possible	Possible	Possible
MOG antibody	Positive	Negative	Negative	Negative	Negative	Negative
AQP4 antibody	Negative	Positive	Negative	Negative	Negative	Negative
GFAP antibody	Negative	Negative	Negative	Negative	Negative	Negative
Oligoclonal bands	Fewer	Fewer	Positive	Fewer	Negative	Negative
TRUST and anti-TP	Negative	Negative	Negative	Negative	Positive	Negative
HIV antibody	Negative	Negative	Negative	Negative	Negative	Positive
MRI						
Optic neuritis	>1/2 optic nerve	>1/2 optic nerve	<1/2 optic nerve	<1/2 optic nerve	Possible	Possible
Transverse myelitis	Possible	>3 vertebral segments	<3 vertebral segments	>3 vertebral segments	>3 vertebral segments	>3 vertebral segments
T2 hyperintensities in brain	Multiple or single white matter lesions	Area postrema surrounding the third and fourth ventricles	Dawson’s finger sign	Massive white matter lesions	Lack of specificity	Lack of specificity
Therapy	Immunosuppressant	Immunosuppressant	Immunosuppressant	Immunosuppressant	Penicillin	Antiretroviral therapy

MOGAD, myelin oligodendrocyte glycoprotein antibody-associated disease; NMOSD, neuromyelitis optica spectrum disorder; MS, multiple sclerosis; ADEM, acute disseminated encephalomyelitis; AIDS, acquired immunodeficiency syndrome; CSF, cerebrospinal fluid; AQP4, aquaporin-4; GFAP, glial fibrillary acidic protein; TRUST, syphilis with serum toluidine red unheated serum test; anti-TP, anti-*Treponema pallidum*-specific antibodies; WBC, white blood cell.

The patient revealed a sharp decline in visual acuity in both eyes, accompanied by afferent pupil defects. Subsequently, the patient developed myelitis and ADEM, which were characterized by impaired consciousness, elevated C-reactive protein levels, raised intracranial pressure, elevated white blood cell and protein levels in the CSF, and abnormal signals in the intracranial region and spinal cord. We noticed a significant increase in C-reactive protein levels in the patient. Sotirios G and Doukas et al. reported a rare case of MOGAD in a 40-year-old patient with COVID-19, and laboratory testing revealed a C-reactive protein level of 67 mg/L (4). The exact pathophysiology of elevated C-reactive protein in these MOGAD patients with syphilis, HIV, or COVID-19 remains unclear. One hypothesis suggests that it is a consequence of specific pathogen infections and MOGAD, which may interact with each other. These infections may activate the immune system, making MOGAD’s autoimmune response more active, thereby jointly promoting an increase in C-reactive protein levels. In the present case, the symptom onset involved optic neuritis accompanied by ADEM. Optic neuritis is characterized by reduced visual acuity and is associated with retrobulbar orbital pain, color vision loss, and afferent pupillary defect (5). Bilateral optic neuritis is the most common clinical subtype of MOGAD, particularly in adults. ADEM is associated with acute polyfocal neurological deficits with encephalopathy and multifocal T2 bright lesions, including the cerebral white matter and gray matter, on MRI (6).

Considering that the patient had a 3-year history of syphilis and AIDS, neurosyphilis and AIDS could have also contributed to fever, disorders of consciousness, and optic neuropathy (7). Differentiating MOGAD from central nervous system damage caused by syphilis and HIV was important. The patient received prior penicillin and antiretroviral therapy for syphilis and HIV, and the abovementioned diseases were well controlled. TRUST in the CSF was negative, no significant decrease in CD4+ T cells was noted, and there was no evidence of secondary or tertiary syphilis and AIDS-related complications. Therefore, we concluded the diagnosis of MOGAD combined with syphilis and AIDS. MOGAD is frequently preceded by infections, including respiratory infections, herpes simplex virus, and Epstein–Barr virus. Molecular mimicry, epitope spreading, and other underlying pathomechanisms may promote the production of MOG antibodies, ultimately leading to white matter demyelination with preserved oligodendrocytes; the infiltration of CD4+ T cells, neutrophils, and macrophages; and complement deposition (8). Given that syphilis and HIV are rarely reported as MOGAD precursor infections, this particular mechanism remains unclear (9–11). Syphilis mainly indirectly weakens the immune response through chronic inflammation, rather than directly damaging immune cells. HIV directly destroys the “command center” of the immune system (CD4+ T cells), leading to global immunodeficiency (12, 13). High-dose corticosteroids are the first-line treatment for the acute phase of MOGAD. Intravenous immunoglobulin and plasma exchange can be attempted in patients with poor efficacy. Although maintenance therapy remains controversial, most scholars believe that low-dose corticosteroids or broad-spectrum immunosuppressants are effective in preventing recurrence (14, 15). Few large cohort studies have reported on the treatment of MOGAD with syphilis and HIV in the previous literature. Based on existing case reports (16), high-dose corticosteroids may increase the risk of infection, leading to poor prognosis and even death. In this case, intravenous immunoglobulins (0.4 g/kg × 5 days) were administered during the acute phase. At the time of discharge, the patient had no fever, his vision was normal, and his consciousness was clear. After a 6-month follow-up, it was found that the patient had a good prognosis.

## 4 Conclusion

Once patients with syphilis and HIV experience neurological symptoms, central nervous system damage caused by neurosyphilis and AIDS should be considered, and MOG/AQP4/GFAP antibodies and oligoclonal bands should be tested to exclude central nervous system demyelinating diseases such as MOGAD, NMOSD, GFAP astrocytopathy, and MS. Early immune intervention is very important, but it needs to balance the rapid suppression of inflammatory reactions and the prevention of infection recurrence. Our case suggests that IVIG may be a good choice. Clinical cohort data are required to better understand the epidemiology, clinical manifestations, prognosis, and outcomes of patients with MOGAD, syphilis, and HIV.

## Data Availability

The datasets presented in this article are not readily available because of ethical and privacy restrictions. Requests to access the datasets should be directed to the corresponding author/s.
